# Repeatability and Reproducibility of Decisions by Latent Fingerprint Examiners

**DOI:** 10.1371/journal.pone.0032800

**Published:** 2012-03-12

**Authors:** Bradford T. Ulery, R. Austin Hicklin, JoAnn Buscaglia, Maria Antonia Roberts

**Affiliations:** 1 Noblis, Falls Church, Virginia, United States of America; 2 Counterterrorism and Forensic Science Research Unit, Federal Bureau of Investigation Laboratory Division, Quantico, Virginia, United States of America; 3 Latent Print Support Unit, Federal Bureau of Investigation Laboratory Division, Quantico, Virginia, United States of America; National Taiwan University, Taiwan

## Abstract

The interpretation of forensic fingerprint evidence relies on the expertise of latent print examiners. We tested latent print examiners on the extent to which they reached consistent decisions. This study assessed intra-examiner repeatability by retesting 72 examiners on comparisons of latent and exemplar fingerprints, after an interval of approximately seven months; each examiner was reassigned 25 image pairs for comparison, out of total pool of 744 image pairs. We compare these repeatability results with reproducibility (inter-examiner) results derived from our previous study. Examiners repeated 89.1% of their individualization decisions, and 90.1% of their exclusion decisions; most of the changed decisions resulted in inconclusive decisions. Repeatability of comparison decisions (individualization, exclusion, inconclusive) was 90.0% for mated pairs, and 85.9% for nonmated pairs. Repeatability and reproducibility were notably lower for comparisons assessed by the examiners as “difficult” than for “easy” or “moderate” comparisons, indicating that examiners' assessments of difficulty may be useful for quality assurance. No false positive errors were repeated (n = 4); 30% of false negative errors were repeated. One percent of latent value decisions were completely reversed (no value even for exclusion vs. of value for individualization). Most of the inter- and intra-examiner variability concerned whether the examiners considered the information available to be sufficient to reach a conclusion; this variability was concentrated on specific image pairs such that repeatability and reproducibility were very high on some comparisons and very low on others. Much of the variability appears to be due to making categorical decisions in borderline cases.

## Introduction

The forensic use of latent fingerprints and palmprints depends on the analysis, comparison, and evaluation decisions made by expert latent print examiners. An assessment of the accuracy and reliability of those decisions is therefore critical to validating the use of latent prints in forensic science [1]: the recipients of latent print examiners' decisions must know whether those decisions are correct, and whether they would get the same decisions on a different occasion.

This study measures repeatability and reproducibility of latent print examiners' decisions: we use the term *reproducibility* to refer to inter-examiner agreement (whether two examiners reach the same decision on the same fingerprints) and *repeatability* to refer to intra-examiner agreement (whether one examiner consistently reaches the same decision on the same fingerprints).

To date, there have been several studies demonstrating that examiner decisions are not always in agreement [2], [3], [4], [5] and that individual examiners sometimes change their decisions [4], [6], [7]. Prior work on repeatability has demonstrated that changed decisions occur under both biasing and non-biasing circumstances; some recent discussion has focused on contextual bias as a potential source of erroneous identifications [8], [9], [6]. In this study, we investigate the repeatability and reproducibility of decisions under test conditions designed to minimize the effects of bias and other contextual influences.

Our previous study [5] evaluated the accuracy and reproducibility of examiners' decisions. Subsequent to that initial test, we retested the original participants to observe whether examiners would repeat their decisions after an interval of seven months. Here we present repeatability data from the retest, and further analyses of the reproducibility data from the initial test, to more completely characterize the accuracy and reliability of latent print examiners.

The results of this study strengthen the understanding of latent examiners' decisions, contributing to the scientific basis for fingerprint examination. This serves the needs of the forensic science community by clarifying the value of forensic evidence with respect to legal questions of admissibility; by helping to identify where to focus training, certification, and standardization; and by providing data to assist agencies in managing finite resources and improving procedures to ensure the quality of results.

### Background

Latent prints (“*latents*”) are friction ridge impressions (fingerprints, palmprints, or footprints) left unintentionally on items such as those found at crime scenes. Exemplar prints (“*exemplars*”), generally of higher quality, are collected under controlled conditions from a known subject using ink on paper or digitally with a livescan device. *Latent print examiners* use their expertise rather than a quantitative standard to determine if the information content is *sufficient* to support a given decision. During analysis of a print, latent print examiners must determine the *value* of the image before proceeding to comparison: value for individualization (*VID*), value for exclusion only (*VEO*), or no value (*NV*). After a comparison of two prints, the examiner makes an evaluation decision of *individualization*, *exclusion*, or *inconclusive*. The VEO category is used operationally by a minority of participating latent print examiners (see *Information S10*). Many agencies combine the VID and VEO categories as “value for comparison” [10].

Latent-exemplar image pairs collected under controlled conditions for research are known to be *mated* (from the same source) or *nonmated* (from different sources). An individualization decision based on mated prints is a *true positive*, but if based on nonmated prints, it is a *false positive* (error); an exclusion decision based on mated prints is a *false negative* (error), but is a *true negative* if based on nonmated prints. The term “error” is used in this paper only in reference to false positive and false negative conclusions when they contradict known ground truth. No such absolute criterion exists for judging whether the evidence supports reaching a conclusion as opposed to making an inconclusive decision. The failure to make an individualization decision on mated prints includes inconclusive decisions as well as false negative errors: such *missed individualizations* may or may not be considered appropriate based on the sufficiency of information available. The best information we have to evaluate the appropriateness of reaching a conclusion is the collective judgments of the experts. Operationally, the reproducibility of a decision by another examiner therefore serves as a surrogate for ground truth regarding the appropriateness of a decision.

This retest was motivated in part by the inter-examiner disagreements and error rates on the initial test [5], summarized here. The overall false positive rate for VID comparisons of nonmated pairs (FPR_VID_) was 0.1%; the false negative rate for all comparisons of mated pairs (FNR_CMP_) was 7.5%. (We use VID and VEO to qualify comparison decisions to indicate that we are referencing specific subsets of the data based on latent value. For example, “VID comparisons” include comparison decisions based on latents assessed as VID, and not those decisions based on latents assessed as VEO.) No two examiners made false positive errors on the same comparison. However, examiners frequently made false negative errors on the same comparison: 85% of examiners committed false negative errors; these were distributed across half of the mated image pairs. False negative rates and conclusion rates varied by individual examiner and by image pair. Inter-examiner agreement at the 90% level (at least 90% of examiners agreeing) was achieved on 66% of latents (deciding whether VID or Not VID); 73% of mated pairs (deciding individualization vs. inconclusive); and 56% of nonmated pairs (deciding exclusion vs. inconclusive). These descriptive statistics pertain specifically to the mix of data and participating examiners included in the initial study. The individual examiners did not (and will not) know how they performed individually on the initial test; the retest was conducted before any results were reported from the initial test.

The initial study demonstrated (consistent with prior expectations) that reproducibility of decisions is highly image dependent. The overall level of reproducibility on a test such as this, or in any specific operational environment, can be expected to reflect the mix of data encountered (image characteristics and the proportion of mated to nonmated pairs) and the mix of examiners (skills). Based on the results of the initial test, we were interested to determine whether erroneous decisions were any less repeatable than correct decisions, which would have operational implications for quality assurance. We were also interested in repeatability from the perspective of the recipient of a decision (posterior probabilities): are certain decisions more or less repeatable than other decisions? We therefore designed the retest and focused the analyses to address these several questions. The rates measured in this study provide useful reference estimates that can inform decision making and guide future research; the results are not representative of all situations, and do not account for operational context and safeguards.

The probability that an examiner will repeat a decision, or that another examiner will reproduce a decision, depends on many factors. One factor is the type of examination performed: for example, whether comparing a single latent to a single exemplar, or multiple latents to full sets of exemplar prints. We can expect repeatability to vary from examiner to examiner, and may expect reproducibility to vary by subpopulation (such as those with similar training, or by organization). We can expect that when the quality and quantity of corresponding information present in a pair of images is either very high or very low, repeatability and reproducibility will be higher than when the information content is marginal or when the examination is complex due to factors such as distortion or background issues. We should not expect equal rates of agreement for individualization decisions as for exclusion decisions for two reasons: an exclusion can be justified based on a single discrepancy, whereas individualization requires sufficient features in agreement to conclude that the two impressions originated from the same source; the mated and nonmated image pairs represent distinct populations whose test samples were selected by distinct procedures. Finally, rates of agreement depend on how agreement is defined, which in turn should reflect the question under investigation: for example, distinguishing inconclusive from individualization is important when asking whether examiners agree as to the sufficiency of the evidence, but this distinction is not relevant when asking whether blind verification has the potential to detect false negative errors.

To date, there has been little empirical research on the repeatability and reproducibility of decisions by latent print examiners. What has been published demonstrates that examiners usually agree, but are not entirely consistent; the relative importance of the various contributing factors has not been established.

## Materials and Methods

(See also *Information S1*)

Ethics Statement: The collection of fingerprints from human subjects was approved by the FBI Laboratory Institutional Review Board and the Noblis Institutional Review Board. Use of latent print examiners in the study was approved by the FBI Laboratory Institutional Review Board, and written informed consent was obtained from all participating examiners.

The repeatability retest used the procedures and fingerprints from the initial study, and a subset of the participants. The examiners were presented with fingerprints they had seen in the initial study; they were not told that they had previously seen these prints. Latents and mated exemplars included a broad range of attributes and quality, within a range typical of casework. Each comparison was of an image pair that consisted of one latent and one exemplar. Image pairs were selected to be challenging: nonmated pairs were based on difficult comparisons resulting from searches of the Federal Bureau of Investigation's Integrated Automated Fingerprint Identification System (IAFIS), which at the time of data selection included exemplars from over 58 million persons with criminal records, or 580 million distinct fingers. A large majority of the participants agreed that the fingerprints were representative of casework [5].

The retest used the custom test software that was developed by Noblis for the initial study. The software presented latent and exemplar images to the participants, allowed a limited amount of image processing, and recorded their decisions. For each image pair, the examiner was asked to determine the value of the latent: VID, VEO, or NV. If the decision was NV, the exemplar was not presented for comparison; otherwise, the exemplar was presented and the examiner was required to make a decision of individualization, exclusion (of the finger), or inconclusive. Examiners were required to perform comparisons in the assigned order and could not revisit previous decisions.

### Repeatability data

Out of the 169 latent print examiners who participated in the initial study, 72 participated in the retest; as in the initial study, most were volunteers, while the others were encouraged or required to participate by their employers. Each examiner was reassigned 25 image pairs that he or she had seen during the initial test, seven months earlier. Data selection was based on stratified sampling according to whether the image pairs were mated or nonmated, and whether the examiner committed an error on the initial test. Examiners were not told how image pairs were selected for the retests, nor were they informed that these were comparisons they had performed earlier. The 25 image pairs were assigned to each examiner as follows (*Information S2*):

- 9 nonmated image pairs. Examiners who had previously committed a false positive error were reassigned that image pair (*FalsePos* dataset, n = 3 decisions). The remainder of these image pairs were selected at random (*RandomNonMates*, n = 645 decisions).
- 16 mated image pairs. These were partitioned in three sets:
  1. 11 were selected at random among image pairs on which the examiner had not committed a false negative error (*RandomMates*, n = 792 decisions);
  2. if the examiner committed any false negative errors, these pairs (up to 5) were selected (*FalseNeg*, n = 226 decisions);
  3. The remaining pairs, if any, were selected at random among those on which the examiner had not committed a false negative error (*ExtraMates*, n = 134 decisions). These were not intended for use in this analysis, in order to prevent over-representing the performance of those examiners who committed fewer false negative errors.

The retest included a total of 339 latents, 389 mated image pairs, and 210 nonmated image pairs (excluding *ExtraMates*).

There were two related sources of data from our tests of these examiners, which provide additional information on the repeatability of latent value decisions, and of false positive and false negative errors:

- The “*Within-test*” dataset provides repeatability data for latent value decisions, where the second decision was made within hours or days of the initial decision (*Information S5*). During the initial test, each of the 169 examiners was assigned approximately 100 image pairs, for a total of 17,121 presentations. Among these there were 900 cases in which an examiner saw the same latent twice.
- The “*Multi42*” dataset provides repeatability data for an additional 42 participants (exclusive of the 72); this data is limited to image pairs on which these examiners made false positive or false negative errors on the initial test. *Multi42* was taken approximately three months after the initial test and followed the same test protocol as the other tests, but was designed for multiple purposes: only the portion of data from this test that pertains to repeatability of errors is reported here. Each examiner was reassigned up to 7 image pairs on which that examiner had made false negative errors (*FalseNeg_M*, n = 105 decisions; 69 mated image pairs); one examiner who initially committed a false positive error was reassigned that image pair (*FalsePos*, n = 1 decision).

In *Information S3*, we compare the performance of the retest participants on three reference measures from the initial test. These measures reveal a notable difference in the false negative rates among the groups: the retest participants had a higher false negative rate (FNR_CMP_ = 8.8%) than the other participants (6.4%).

### Reproducibility data

The reproducibility data comes from the initial test on which each examiner was assigned approximately 100 image pairs. For comparability, reproducibility data is limited to responses by the 72 examiners who participated in the retest.

### Agreement statistics

We use percentage agreement, 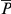, to describe both intra-examiner agreement (repeatability) and inter-examiner agreement (reproducibility). This commonly used statistic simply describes the proportion of times paired responses are in agreement – either multiple raters on the same test item in the case of reproducibility, or the same rater in the case of repeatability. A confidence interval for this metric can be accomplished by bootstrapping [11].

Several other commonly reported measures of rater agreement are not purely descriptive, because they introduce modeling assumptions either explicitly or implicitly. Fleiss's kappa [12] corrects the pure, descriptive metric to account for agreements due to chance. There has been much discussion of the issues involved in making such a correction (e.g., [13], [14], [15]). At the very least, a realistic correction for chance requires some modeling of randomness in the decision process, and the resulting metric is no longer purely descriptive. In *Information S8*), we report results using one such metric, *kappa_N_* [16], in a summary of the main results.

Rather than attempting to model randomness in the decision process, one can model the classification process itself. There is a considerable amount of literature on models in which the observed ratings are partially determined by unobservables that themselves have been randomly sampled. The beta-binomial distribution results from a particularly simple model, under which each item (e.g., image-pair) has associated with it an unobservable probability of being classified as “A” instead of “B”. Shuckers [17] discusses its use in fingerprint examination analysis. In *Information S9*, we use the beta-binomial distribution to derive confidence intervals as a means of providing some indication of our measurement precision.

A much larger class of models fall under the rubric Latent Structure Analysis. Uebersax [18] reviews these models in the context of agreement analysis. For example, under one such model, each item takes on a value for an unobservable continuous variable. Each rater has his/her unobservable threshold for this variable, which determines the rater's personal probability of assigning a classification to the item. Among other advantages, this enables estimating the distribution of these thresholds.

Percentage agreement (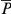) is defined as follows. Let *P_i_* represent the extent of agreement on the *i* ^th^ image (or image pair):
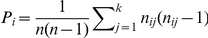
where *n* is the number of decisions, *k* is the number of decision categories, and *n_ij_* is the number of decisions assigning the *i* ^th^ image (or image pair) to the *j* ^th^ category. *P_i_* is a proportion and can take on values from 0 to 1. When calculating reproducibility, *n* represents the number of examiners deciding on the *i^th^* image (or image pair). When calculating repeatability, *n* = 2, representing the test and retest decisions made by one examiner.


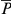
 is simply the mean agreement over a set of *N* test questions (images or image pairs):
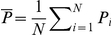
This measure weights each test question (image or image pair) equally. Similar results derived from the contingency tables presented would implicitly weight each response equally, resulting in slightly different values.

Both 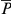 and *kappa* implicitly treat all disagreements as being equally serious. So, for example, the disagreement “individualization vs. exclusion” is not weighted differently than the disagreement “individualization vs. inconclusive.” Because various types of disagreements have very different operational consequences, we report separate statistics for each by applying the percentage agreement statistic in multiple ways to address distinct research questions. For example, we measure agreement based on population (mates vs. nonmates), and decision granularity (e.g. 2-way decisions such as {VID, not VID} vs. 3-way decisions such as {VID, VEO, NV}). It is important to recognize that chance alone would account for some level of agreement: as with any true/false test, the percentage agreement would be expected to be substantially greater than zero even if examiners were guessing. When the response frequency is unequal among the categories, we expect a higher level of agreement; when there are more categories, we expect a lower level of agreement.

## Results

We report intra-examiner (repeatability) results and compare them to the inter-examiner (reproducibility) results from the initial test. The results include analyses of latent value decisions, comparison decisions, and comparison difficulty. Because the relative proportions of mated and nonmated image pairs are test-specific, comparison decisions are reported separately for mated and nonmated data. Except where specific reference is made to the *Within-test* dataset or the *Multi42* dataset, the repeatability results are based on the main retest (72 examiners); all reproducibility statistics are from the initial test, and are limited to those 72 retest participants for comparability.

The responses provided on these tests were decisions of individual examiners, which may not reflect the final decisions that an agency would have reported with the benefit of organizational quality management (e.g., verification, or technical and administrative reviews).

### Analysis of latent value

**Figure 1:**
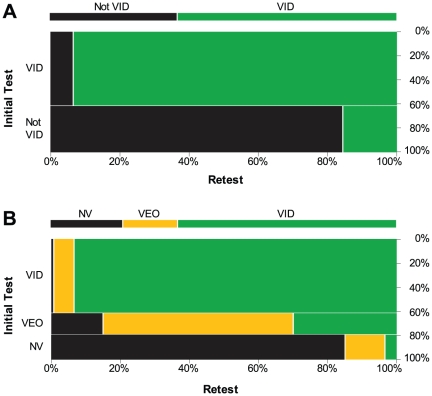
Test-retest repeatability of latent value decisions (mosaic charts). (A) 2-way {VID, Not VID} latent value decisions: 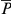 = 89.7%. (B) 3-way latent value decisions {NV, VEO, VID} including category “value for exclusion only”: 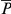 = 84.6%. These mosaic plots depict the tabular data from Table 1, indicating for each category of initial test response (y-axis) the proportion of each category of retest response (x-axis).

Examiners determined the value of each latent print before proceeding to comparison. Together, the initial test and retest resulted in 1,403 pairs of intra-examiner latent value decisions among randomly selected latents that were assigned twice to the same examiner (latents from the *RandomMates* and *RandomNonMates* datasets; see *Information S6*) for further discussion of data selection for latent value analyses). The extent of repeatability depends on the number of decision categories, based on the treatment of the category “value for exclusion only”. On the question of whether a latent was of value for individualization (2-way decision: {VID, not VID}), repeatability of initial responses was 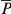 = 89.7% (Fig. 1A). When examiners were required to further differentiate NV from VEO (3-way decision: {VID, VEO, NV}), repeatability dropped to 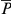 = 84.6% (Fig. 1B). Complete reversals (between NV and VID) occurred at the rate of 1%. The charts in Fig. 1 depict the contingency table of examiner value decisions (Table 1) as mosaic plots, where the area of each colored region represents the proportion of a combination of initial and retest decisions. For example, in Fig. 1A, 61% of initial value decisions on latents were VID; this corresponds to the height of the row labeled “VID.” On retest, 93% of those VID decisions were repeated; hence, 93% of that row is colored green to indicate VID decisions on the retest. Reading across any one row of a mosaic reveals the conditional probability of a second response given the initial response.

**Table 1:** Test-retest repeatability of latent value decisions (3-way contingency table).

	Retest				
Initial Test	NV	VEO	VID	Total	Repeated
NV	249	34	10	293	85%
VEO	38	137	75	250	55%
VID	8	51	801	860	93%
Total	295	222	886	1,403	

The table summarizes 1,403 pairs of decisions made by 72 examiners on 339 distinct latent images. Examiners changed their 2-way {VID, not VID} latent value decisions on 94 distinct latents.

Table 1 reveals two asymmetries. Examiners appeared slightly more willing to call latents VID on the retest than on the initial test, with most of the shift from VEO to VID. There is also a conditional asymmetry resulting from the fact that VEO is an intermediate decision category. Many latents were inarguably VID and NV decisions, and therefore were much more stable than VEO (no latents were unanimously VEO): 85% of NV decisions and 93% of VID decisions on the initial test were repeated, whereas only 55% of VEO decisions were repeated.

The *Within-test* repeatability data showed very similar results when examiners were retested over a period of days (median 7 days) rather than months (*Information S5*). On the question of whether a latent was of value for individualization, repeatability was 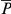 = 92.2%. This rate is only slightly higher (*p* = 0.026, one-sided) than the rate measured on the retest (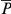 = 89.7%). When examiners were required to further differentiate NV from VEO, repeatability dropped to 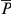 = 88.8%. Complete reversals (between NV and VID) occurred at the rate of 1%.

Reproducibility of VID decisions was unanimous on 42% of the latents. The extent of unanimity reflects the data selection: this test was designed to focus on difficult image pairs; if the test had included more latents that were obviously of value or obviously of no value, the overall reproducibility of value decisions would have been higher.

**Figure 2:**
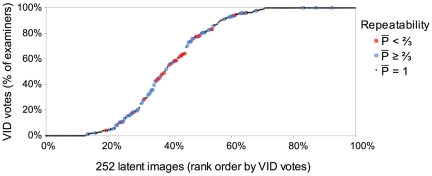
Repeatability and reproducibility of 2-way latent value decisions {VID vs. Not VID}. Percentage of examiners rating each latent VID (y-axis), in rank order (x-axis), color-coded by repeatability; n = 252 latents on which at least 3 examiners were retested. Examiners were initially unanimous on 107 of these 252 latents; value decisions changed on 3 of these. Reproducibility rates were based on 53.2 mean examiners per latent (s.d. 21.7); repeatability rates were based on 5.0 mean examiners per latent (s.d. 2.3).

Changed value decisions were almost entirely restricted to latents on which there was some disagreement among examiners (Fig. 2). On the retest, changed decisions occurred on nearly half of the latents on which there was not unanimous agreement among examiners (mean of 5.0 retest decisions per latent). Among the 197 retested images on which there was not initially unanimous agreement, repeatability was 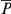 = 83.3%; on these same 197 images, reproducibility was 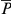 = 75.2%. This association demonstrates that in almost all cases, the specific images on which examiners individually were not consistent in their own decisions also resulted in disagreement among examiners.

On the initial test, some comparisons resulted in individualization decisions (true positives) even though the latent value decision was VEO, for a rate of 1.8% (40 out of 2,220 VEO comparisons of mated pairs). The retest yielded similar results, albeit on a much smaller sample: 4/142 VEO comparisons of mated pairs resulted in individualization decisions (true positives).

### Comparison Decisions

Repeatability on the *RandomNonMates* dataset (Table 2), based on three decision categories {VID individualization, exclusion, no conclusion}, was 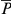 = 85.9%: 90.6% of (true) exclusion decisions were repeated; 73.1% of no conclusion decisions were repeated. We should not expect the proportion of exclusion decisions repeated to equal the proportion of no conclusion decisions repeated: some image pairs will result in more consistent decisions than others, and the test was not designed to result in equal proportions of exclusion, no value or inconclusive decisions. Repeatability on the *RandomMates* dataset (Table 3), based on the same three decision categories, was 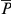 = 90.3%; 89.1% of VID individualization decisions were repeated; 90.9% of no conclusion decisions were repeated. Most of the difference in the repeatability of no conclusion decisions between the *RandomNonMates* and *RandomMates* sample populations may be explained by the fact that the *RandomMates* dataset included a much higher proportion of poor-quality images than did the *RandomNonMates* [5]. We do not report an overall repeatability percentage: because the study design was based on stratified partitions of data, any such overall rate would reflect the relative sizes of the partitions, not any meaningful result.

**Table 2:** Repeatability of comparison decisions on *RandomNonMates* dataset.

	Retest (Nonmates)				
Initial Test	No Conclusion	Exclusion	VID Indiv.	Total	Repeated
No Conclusion	128	47	0	175	73.1%
Exclusion	44	426	0	470	90.6%
Total	172	473	0	645	

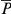
 = 85.9%. Contingency table of the 645 repeat assignments of nonmated image pairs, on which the examiner did not initially commit a false positive error. No conclusion includes NV, inconclusive, and VEO individualization. Exclusions include comparisons of latents rated VEO and VID.

**Table 3:** Repeatability of comparison decisions on *RandomMates* dataset.

	Retest (Mates)				
Initial Test	No Conclusion	Exclusion	VID Indiv.	Total	Repeated
No Conclusion	479	15	33	527	90.9%
VID Indiv.	20	9	236	265	89.1%
Total	499	24	269	792	

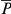
 = 90.3%. Contingency table of the 792 repeat assignments of mated image pairs, on which the examiner did not initially commit a false negative error. Examiners repeated 89.1% of true individualization decisions. No conclusion includes NV, inconclusive, and VEO individualization. Exclusions include comparisons of latents rated VEO and VID.

**Figure 3:**
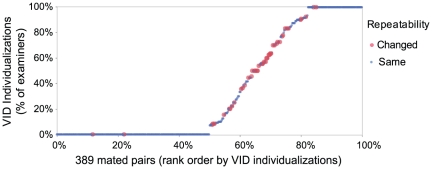
Repeatability and reproducibility of 2-way individualization decisions {VID individualization, other}. Percentage of examiners individualizing mated image pairs (y-axis), in rank order by VID individualization (x-axis), colored-coded by repeatability. Y-axis is based on 4,006 initial decisions (excludes false negative responses; 10.3 mean examiners per image pair; s.d. 2.6). Color-coding is based on 792 retest decisions on 389 mated image pairs (*RandomMates* dataset; 2.0 mean examiners per image pair; s.d. 1.1). Non-repeated decisions occurred on 46 of the 389 image pairs. Examiners were initially unanimous on 257 of the 389; decisions were not repeated on 2 of these.

In those cases where examiners changed their decisions on whether or not there was sufficient information to individualize, such changes almost always occurred on those image pairs that resulted in non-reproduced decisions (Fig. 3); results are very similar to those shown for value decisions (Fig. 2). The majority of decisions that were not repeated changed to or from inconclusive or VEO decisions: most of the intra-examiner inconsistency was with respect to sufficiency to make a conclusion.

As illustrated in Fig. 2 and Fig. 3, many images and image pairs are associated with highly reliable decisions (repeatable and reproducible); among those images (image pairs) where the group does not achieve highly reproducible results, we observe a high level of intra-examiner inconsistency. Much of the lack of reproducibility is associated with decisions that individual examiners do not reliably repeat.

**Figure 4:**
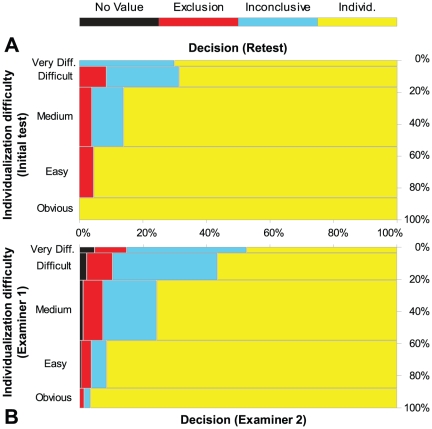
Repeatability (A) and reproducibility (B) of individualization decisions by difficulty. (A) Retest decisions by difficulty where the initial test decision was an individualization (269 paired decisions (test-retest) on 147 image pairs, 144 of which were mated). (B) Reproducibility of individualization decisions by difficulty (1,615 individualization decisions (15,990 paired examiner responses) by the 72 examiners on 249 image pairs, 246 of which were mated). Results for exclusion decisions were similar (*Information S7*).

Examiners were asked to indicate the difficulty of each comparison performed on a scale from “obvious” to “very difficult”. Difficulty proved to be a good predictor of decreased repeatability and reproducibility of both individualization and exclusion decisions (Fig. 4; Table 4; see also *Information S7*).

**Table 4:** Repeatability and reproducibility of individualization and exclusion decisions, by examiner assessment of difficulty.

	Individualization		Exclusion	
	Repeated	Reproduced	Repeated	Reproduced
Obvious/Easy/Medium	92%	85%	88%	77%
Difficult/Very Difficult	69%	55%	70%	50%

**Figure 5:**
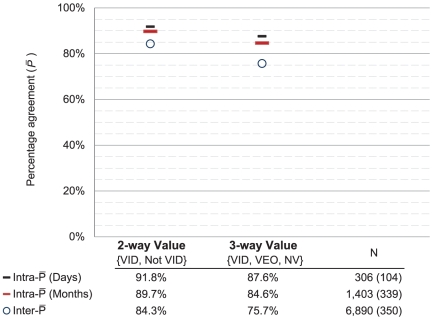
Percentage agreement on latent value. 2-way {VID, Not VID} and 3-way {NV, VEO, VID} latent value repeatability is measured within the initial test (“Days”), and between the test and retest (“Months”). Reproducibility is computed from the initial test results. All statistics are limited to the 72 retest participants; “N” indicates the number of decisions and, parenthetically, the number of distinct latents. Confidence intervals for these estimates are discussed in *Information S9*.

**Figure 6:**
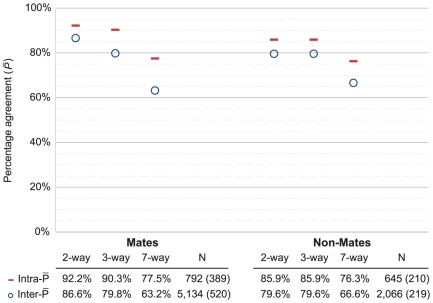
Percentage agreement on comparisons of mated and nonmated image pairs. 2-way Mates {VID individualization, other}, 2-way Nonmates {exclusion, other}, 3-way {VID individualization, any exclusion, other}, and 7-way {NV, VEO inconclusive, VEO exclusion, VEO individualization, VID inconclusive, VID exclusion, VID individualization}. Repeatability is computed from the *RandomMates* and *RandomNonMates* datasets; reproducibility is computed from the initial test results. While the 2-way and 3-way decisions correspond to common operational practice, only a subset of the 7-way distinctions would correspond to any specific operational practice. All statistics are limited to the 72 retest participants; “N” indicates the number of decisions and, parenthetically, the number of distinct image pairs. Confidence intervals for these estimates are discussed in *Information S9*).

Fig. 5 and Fig. 6 summarize the aforementioned repeatability statistics and contrast these with corresponding measures of reproducibility to reveal the broad trends (see also *Information S8*). As expected, we see that agreement decreases as the number of decision categories increases. On latent value decisions, most of the intra-examiner variability was already evident on the *Within-test* dataset; the additional seven months added only a small increment. Fig. 5 and Fig. 6 also show that most of the observed inter-examiner variability can be attributed to intra-examiner effects. This pattern is especially strong on nonmate decisions (Fig. 6, Nonmates), where the intra-examiner rate of disagreement is about 70% as large as the inter-examiner rate of disagreement. On comparisons of mated pairs, intra-examiner effects account for most of the observed variability on individualization decisions (Fig. 6, 2-way Mates), while false negative errors are a major source of inter-examiner disagreements (compare 2-way Mates to 3-way Mates).

**Figure 7:**
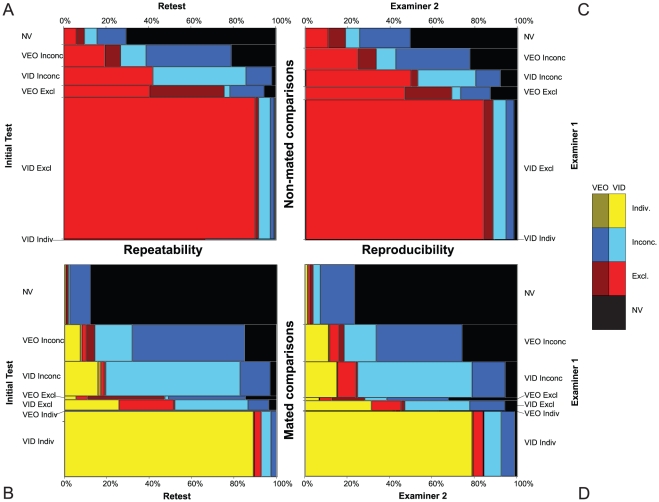
Mosaic displays of 7-way contingency tables for repeatability and reproducibility of examiner decisions. (A) repeatability of nonmated comparison decisions (648 test-retest decision pairs on 210 nonmated pairs); (B) repeatability on mated comparisons (1,018 test-retest decision pairs on 436 mated pairs); (C) reproducibility on nonmated comparisons (19,025 inter-examiner decision pairs derived from 2,066 decisions on 219 nonmated pairs); (D) reproducibility on mated comparisons (51,380 inter-examiner decision pairs derived from 5,134 decisions on 499 mated pairs). The corresponding contingency tables are presented in *Information S4*. Chart B is adjusted to correct for the disproportionate number of false negative errors that were deliberately included in the retest: the height of the exclusion rows was reweighted to correspond to the proportions occurring on the initial test (6.3% of mated pairs in the initial test were false negatives, vs. 13.6% selected for the retest).

The overall patterns of agreement and disagreement tended to be similar for intra- and inter-examiner pairs of responses (Fig. 7; see also *Information S4*). In Fig. 7, mosaics A and B show in detail the patterns of repeatability on nonmated and mated pairs, respectively, for 7-way decisions {NV, VEO inconclusive, VEO exclusion, VEO individualization, VID inconclusive, VID exclusion, VID individualization}. The corresponding patterns for reproducibility (C and D) are quite similar, but the rates of inter-examiner disagreement are higher than the rates of intra-examiner disagreement.

One half of the 3-way disagreements {VID individualization, any exclusion, other} on mated pairs were due to false negative errors (Fig. 7D): 9.6% of the paired responses among examiners were disagreements involving false negative errors.

### Repeatability and Reproducibility of Errors

For the purposes of operational quality assurance, there is a particular interest in understanding repeatability and reproducibility with respect to false positive and false negative errors.

Six false positives were committed by five examiners on the initial test (Table 5): none of these errors were reproduced in the initial test, and none were repeated in the retest (n = 4). No new false positive errors were committed during the retest among 645 randomly selected nonmate repeat assignments (Table 2), which is consistent with the false positive rate of 0.1% on the initial test.

**Table 5:** Examiner responses on the six image pairs (labeled A–F in [5]) that resulted in false positive errors.

		Image Pair (Nonmates)					
Test Response		A	B	C	D	E	F
VEO	Exclusion	2	–	–	–	–	–
	Inconclusive	11*^(R)^*	–	–	–	–	–
	Individualization	–	–	–	–	–	–
VID	Exclusion	6	24*^(R)^*	22	21	20*^(R)^*	21
	Inconclusive	6	5	3	–	1	1*^(R)^*
	Individualization	1*^(I)^*	1*^(I)^*	1*^(I)^*	1*^(I)^*	1*^(I)^*	1*^(I)^*

*Multi42* dataset.

Cell counts indicate the distribution of responses from all 169 examiners on the initial test. The initial (I) and retest (R) responses are indicated for the examiners who committed the false positive errors. One examiner who committed two errors (image pairs C and D) did not participate in the retests; the retest response for one image pair is from the.

The retest participants committed false negative errors at the rate of 8.8% (FNR_CMP_) on the initial test. The majority of those errors were not repeated (Table 6): of the 226 false negative errors that were retested, 68 were repeated (30.1%). We estimate the probability that another examiner would reproduce one of these errors to be 19% (Table 7). We understand these comparative results as follows: “self-verification” (several months later) detected 69.9% of the false negative errors, whereas independent examination of the same images by another examiner (analogous to blind verification) would have detected an estimated 81%. Interestingly, much of the relative benefit of blind verification over this type of self-verification relates to the wide variability in FNR by examiner: false negative errors are produced disproportionately by those examiners with high FNRs, so another examiner selected at random to perform verification is likely to have a lower FNR. Difficulty was not predictive of whether false negative errors would be repeated; the data suggest that greater difficulty is weakly associated with lower reproducibility for false negative errors. Although most errors were not repeated on the retest, examiners did introduce new false negative errors (Table 3). After correcting for the difference in test mix between the initial test and the retest, no significant net change in false negative error rate was observed.

**Table 6:** Repeatability of false negative errors on (A) *FalseNeg and (B) FalseNeg_M* datasets.

A	Retest (Mates)					
Initial Test	Exclusion	Inconclusive	Indiv.	NV	Total	Repeated
Exclusion	68	97	47	14	226	30.1%

B	Multi42 (Mates)					
Initial Test	Exclusion	Inconclusive	Indiv.	NV	Total	Repeated
Exclusion	29	37	23	16	105	27.6%

Data limited to mated pairs that were erroneously excluded in the initial test; includes comparisons of latents rated VEO and VID.

**Table 7:** Repeatability and reproducibility for mated pairs, contingent upon whether the initial decision was false negative.

	Repeatability (Retests)	Reproducibility (Initial Test)
FN (n = 226)	30.1%	19.2%
Not FN (n = 792)	97.0%	94.5%

For comparability, all estimates are limited to responses of the retest participants. “Not FN” includes NV, inconclusive, and individualization. Confidence intervals for these estimates are discussed in *Information S9*, as well as modeling assumptions for the reproducibility results.

Table 7 compares the repeatability and reproducibility rates for mated pairs contingent upon whether the initial decision was an erroneous exclusion. These data indicate that blind verification (estimated by reproducibility) is more effective than self-verification (repeatability) in detecting false negative errors (81% vs. 69.9%). Based on a baseline FNR_CMP_ = 8.8% (as measured among retest participants on the initial test) and Table 7 (first row), we estimate that if every exclusion decision were verified, the resulting rate of erroneously corroborated false negatives would be 2.7% (self-verified) and 1.7% (blind-verified).

## Discussion

In order to better understand limitations to the reliability of examiner decisions, and to develop strategies for improvement, we need to understand the types of errors and disagreements that occur and the circumstances under which they occur. Analyses of repeatability and reproducibility can provide indications of the causal factors contributing to disagreements among examiners and erroneous conclusions. For example, differences in examiner skill or judgment would be consistent with errors and disagreements that tend to persist, whereas differences that do not persist might reflect inadvertent errors or borderline decisions.

While the rates we report reflect the specific test data and the performance of participants, several general conclusions may be drawn. Most but not all examiner decisions were highly repeatable and reproducible. The overall patterns of agreement and disagreement tended to be similar for repeatability and reproducibility. Much of the lack of reproducibility was associated with specific images and image pairs on which individual examiners were not highly consistent. Most of the inter- and intra-examiner inconsistency was with respect to whether the information available was sufficient to make a conclusion. Examiner assessments of comparison difficulty were a good predictor of low repeatability and reproducibility.

Why do examiners not always repeat their own decisions? Most of the inconsistency pertains to whether the examiners considered that the information available was sufficient to reach a conclusion (such as between individualization and inconclusive decisions). Our interpretation is that there is a continuum of the quality and quantity of features as interpreted by examiners. Much of the variability arises from making discrete decisions in this continuous decision space in borderline or complex cases (“complex” decisions are defined in [10]). When decisions were not repeated or reproduced, the majority changed to or from inconclusive or VEO decisions. Lack of repeatability for complex or borderline decisions may be attributed to differences in the examiner's assessments of features in each print, or to differences in how the examiner uses those features in making value or comparison decisions. An examiner's assessments of the quality and quantity of features in a given print may vary. Schiffer and Champod [19] found that the number of minutiae detected by an examiner increases with training; Dror, et al. [20] found that the number of minutiae observed by an examiner varied from test to retest. Differences in assessments of features may be especially critical when key features are ambiguous. However, if the examiner reaches a different decision without changing assessments of the quality and quantity of features, then the examiner is not applying decision criteria consistently. This may be attributable in part to the lack of quantitative criteria and limited qualitative criteria for decisions: in some difficult cases it is not apparent to the examiner whether a conclusion or inconclusive decision is appropriate. Other plausible explanations for why decisions would not be repeated may include inadvertent mistakes, changes in outside influence or bias, or changes in expertise over time. While any of these may apply to casework, given the study design we do not consider contextual bias and changes in expertise to be significant contributing factors to the findings in this study.

Why do different examiners reach different decisions? Much of the observed lack of reproducibility is associated with prints on which individual examiners were not consistent, rather than persistent differences among examiners. When inter-examiner disagreements on decisions are not associated with a lack of repeatability, we suggest the following explanations: examiners differ as to which features are present in each print [9], [19], [20]; examiners differ on the relative costs or implications of decisions (e.g., weighing the benefit of a true positive against the cost of a false positive, or against the cost of an inappropriate inconclusive decision); examiners differ as to whether the information present is sufficient to support a specific decision, while agreeing on features and costs; examiners differ in skill and experience (e.g., we previously found that conclusion rate increased with experience [5]); or examiners differ in their use of terminology (the exact meaning of a decision varies by agency, often related to variations in operating procedures).

Repeatability and reproducibility are of particular importance with respect to false positive and false negative errors. Six false positive errors were committed on the initial test. None of these were reproduced, implying that blind verification should be highly effective at detecting such errors. Four of these comparisons were performed again months later by the examiners who initially committed each error; none of the errors were repeated. The lack of both repeatability and reproducibility suggests that quality control procedures would detect false positive errors such as these, assuming that contradictory decisions would be subject to a rigorous review.

False negative errors contributed substantially to both inter- and intra-examiner disagreements on mated comparisons. The false negative error rate (FNR_CMP_, among the retest participants on the initial test) was 8.8%. When these examiners were retested months later, 69.9% of false negative errors were not repeated. Our corresponding estimate of reproducibility indicates that independent examinations (analogous to blind verification) would have resulted in disagreements on 81% of the false negative errors committed by the original examiner, presumably resulting in a conflict resolution review. This implies that blind verification by another examiner should be expected to catch the majority of false negative errors, but a substantial proportion (19%) would not result in a contradictory decision, and therefore would be corroborated rather than detected. Interestingly, the effectiveness of blind verification is partly due to the wide variability in FNR by examiner: false negative errors are produced disproportionately by those examiners with high FNRs, so another examiner selected at random to perform blind verification is likely to have a lower FNR. While false negative errors were associated with examiner assessments of difficulty [5], the *repeatability* of errors was not well predicted by examiner assessments of difficulty, and repeated false negative errors were not highly concentrated on specific image pairs.

Repeatability and reproducibility are useful surrogate measures of the appropriateness of decisions when there is no “correct” decision, as when deciding between individualization and inconclusive. The reproducibility of decisions has operational relevance in situations where more than one examiner makes a decision on the same prints. Reproducibility as assessed in our study can be seen as an estimate of the effects of *blind* verification [21] – not consulting or non-blind verification. Verification is an agency-specific quality assurance measure conducted with the intent of detecting any errors before decisions are formally reported by the agency. Verification of individualization decisions is standard practice [10], but whether other decisions are verified varies by agency. Typically, the verifier is aware of the first examiner's decision (“non-blind verification”). Blind verification, in which the verifier performs an independent examination without knowledge of the first examiner's decision, is practiced by some agencies either in addition to or instead of non-blind verification. In casework, examiners also may consult with each other, benefiting from a second opinion prior to reaching a decision. The repeatability of decisions has a more subtle relation to casework: in practice, examiners typically have hours or days to catch any mistakes and reassess complex decisions before reporting them. Our study did not provide an opportunity for examiners to reconsider their decisions at a later time before making a final decision, and therefore might underestimate the repeatability of decisions in practice.

Our estimates of reproducibility and repeatability may differ from operations for several reasons. The comparisons in the test were selected to be representative of difficult comparisons from searches of an Automated Fingerprint Identification System (AFIS), including few comparisons where the correct conclusion was obvious. The responses provided on this test were decisions of individual examiners without the benefit of verification or quality assurance, and therefore may not correspond to the final decisions reported by an agency. Examiners also were not permitted to revisit their own decisions during the test. Because practices vary from agency to agency, the test required some examiners to make distinctions that may have been unfamiliar, or at least outside their routine practice. Because participants knew that they were being tested, some may have reacted to the test (“Hawthorne effect”) by trying harder than usual to reach conclusions, or by being more or less cautious than during casework.

Many of the issues raised by these findings could be addressed through enhancements to quality management systems such as the following, some of which are currently used in some forensic laboratories:

- Blind verification can be expected to be effective in detecting most errors and flagging debatable decisions, and should not be limited to individualization decisions.
- Examiner assessments of difficulty may be useful in targeted quality control, which could focus on difficult decisions: operating procedures could provide means for an examiner to indicate when a particular decision is complex. Quality control measures, however, should not focus solely on difficult decisions, since even easy or obvious decisions were not always repeated or reproduced.
- Borderline and complex decisions may benefit from collaboration and consultation among examiners to take advantage of inter-examiner variation on feature selection or decision thresholds.
- Metrics derived from the quality and quantity of features used in making a decision may assist examiners in preventing mistakes, and in making appropriate decisions in complex comparisons. Such metrics may be used to flag complex decisions that should go through additional quality assurance review and in arbitration of disagreements between examiners.
- Errors detected in casework could provide prints for use in training; these could be analyzed to determine the attributes of data and the individuals associated with these errors. This permits training to be targeted for individual examiners; in addition, training for all examiners can be based on lessons learned from specific errors. Missed individualizations may be addressed through a continual improvement process similar to that indicated for errors.
- Procedures for detailed documentation of the features used in analysis or comparison decisions could be used to assist in arbitrating inter-examiner disagreements at the feature level.
- Human factors analyses may be used to identify issues contributing to errors or a lack of repeatability and reproducibility; these analyses would focus on areas such as software user interfaces, potential sources of bias, or uniform understanding of procedures.

There is a need for dialog in the community to address the extensive differences in terminology and procedures in the latent print community (see survey responses in [5]). For example, the relatively high FNR suggests the need to come to agreement on appropriate criteria for exclusion decisions, including decision thresholds based on costs and operational implications.

Further research is needed to better understand how inter- and intra-examiner variability arises. One approach to understanding the source of inter-examiner disagreements would be to conduct a “white box” test in which examiners document the basis for their decisions in the form of image markup. The objectives of such a study would be to investigate similarities and differences in examiners' interpretations of the features in a latent; how examiners assess sufficiency to reach a conclusion; and to assist in the development of guidelines and automated metrics to use the quality and quantity of features in an image to predict whether a decision is likely to be debatable or highly reproducible.

## Supporting Information

Information S1 **Overview of initial study design.**
(PDF)

Information S2 **Structure of the retest.**
(PDF)

Information S3 **Representativeness of retest participants.**
(PDF)

Information S4 **Repeatability and reproducibility contingency tables.**
(PDF)

Information S5 **Within-test repeatability of value decisions.**
(PDF)

Information S6 **Data used in the latent value repeatability analysis.**
(PDF)

Information S7 **Difficulty predicts repeatability and reproducibility.**
(PDF)

Information S8 **Summary agreement statistics.**
(PDF)

Information S9 **Confidence intervals.**
(PDF)

Information S10 **Use of “Value for Exclusion Only” category.**
(PDF)
