# Supplementary material for: Repeatability and Reproducibility of Decisions by Latent Fingerprint Examiners
Source: PLoS One. 2012 Mar 12;7(3):e32800. doi: 10.1371/journal.pone.0032800 (PMC3299696; doi:10.1371/journal.pone.0032800)
Supplement: Information S1 — Overview of initial study design. (PDF) [file pone.0032800.s001.pdf]

## Overview of initial study design

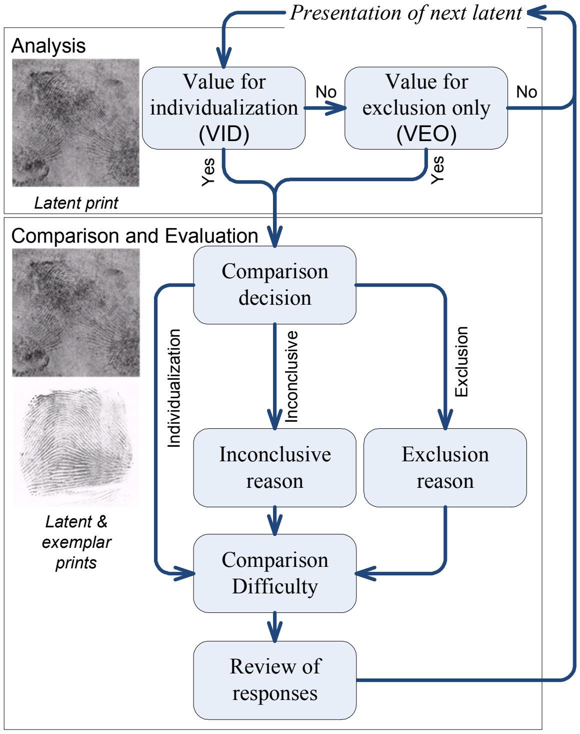


Fig. S1a: Software workflow used in all tests. Each examiner was assigned a distinct, randomized sequence of image pairs. For each pair, the latent was presented first for a value decision; if it was determined to be no value, the test proceeded directly to the latent from the next image pair; otherwise an exemplar was presented for comparison and evaluation. Previously published. [1]

Participation was open to practicing latent print examiners from across the fingerprint community. A total of 169 latent print examiners participated; most were volunteers, while the others were encouraged or required to participate by their employers. Participants were diverse with respect to organization, training history, and other factors. The latent print examiners were generally highly experienced: median experience was 10 years, and 83% were certified as latent print examiners. More detailed descriptions of participants, fingerprint data, and study procedures are included in the Appendix from the initial study [1].

The fingerprint data included 356 latents, from 165 distinct fingers from 21 people, and 484 exemplars. These were combined to form 744 distinct latent-exemplar image pairs. There were 520 mated and 224 nonmated pairs.

We sought diversity in fingerprint data, within a range typical of casework. Subject matter experts selected the latents and mated exemplars from a much larger pool of images to include a broad range of attributes and quality. Latents of low quality were included in the study to evaluate the consensus among examiners in making value decisions about difficult latents. The exemplar data included a larger proportion of poor-quality exemplars than would be representative of exemplars from the FBI's Integrated AFIS (IAFIS). Image pairs were selected to be challenging. Mated pairs were randomly selected from the multiple latents and exemplars available for each finger position. Nonmated pairs were based on difficult comparisons resulting from searches of IAFIS; at the time of data collection, IAFIS included exemplars from over 58 million persons with criminal records, or 580 million distinct fingers; the size of the IAFIS database continues to grow. Participants were surveyed and a large majority of the respondents agreed that the data were representative of casework.

---

***References***

1. Ulery BT, Hicklin RA, Buscaglia J, Roberts MA (2011) Accuracy and reliability of forensic latent fingerprint decisions. Proc Natl Acad Sci USA 108(19): 7733-7738. Available: http://www.pnas.org/content/108/19/7733.full.pdf
