# Supplementary material for: Repeatability and Reproducibility of Decisions by Latent Fingerprint Examiners
Source: PLoS One. 2012 Mar 12;7(3):e32800. doi: 10.1371/journal.pone.0032800 (PMC3299696; doi:10.1371/journal.pone.0032800)
Supplement: Information S2 — Structure of the retest. (PDF) [file pone.0032800.s002.pdf]

Structure of retest

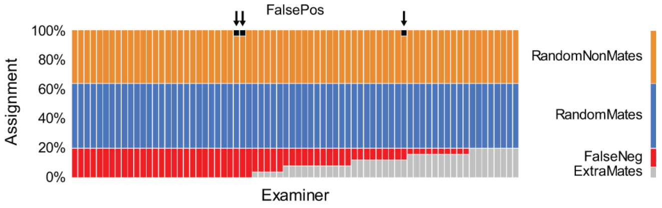

Fig. S2a: Structure of the retest. Each examiner was assigned 25 image pairs selected from among the 100 image pairs previously assigned to that examiner in the initial test. The three erroneous individualizations in the retest are shown in black.
