# Supplementary material for: Repeatability and Reproducibility of Decisions by Latent Fingerprint Examiners
Source: PLoS One. 2012 Mar 12;7(3):e32800. doi: 10.1371/journal.pone.0032800 (PMC3299696; doi:10.1371/journal.pone.0032800)
Supplement: Information S3 — Representativeness of retest participants. (PDF) [file pone.0032800.s003.pdf]

Representativeness of retest participants

	FNR_{CMP}	TPR_{PRES}	TNR_{PRES}
Retest (72 examiners)	8.8%	31.4%	72.9%
Multi42 (42 examiners)	6.3%	34.0%	71.8%
Not retested (55 examiners)	6.4%	31.4%	68.2%
Initial test (169 examiners)	7.5%	32.0%	71.2%

Table S3a: Representativeness of the retest participants with respect to the 169 participants whose performance results were previously published. Three rates are shown for each group: FNR_{CMP}, false negative rate among all comparisons performed; TPR_{PRES}, true positive rate among all presentations of mated pairs; and TNR_{PRES}, true negative rate among all presentations of nonmated pairs. Retest participants had a notably higher false negative rate than those who were not retested. Assignment of participants to the retest or the multiple purpose test (*Multi42*) was based on geographic location.
