# Supplementary material for: Repeatability and Reproducibility of Decisions by Latent Fingerprint Examiners
Source: PLoS One. 2012 Mar 12;7(3):e32800. doi: 10.1371/journal.pone.0032800 (PMC3299696; doi:10.1371/journal.pone.0032800)
Supplement: Information S4 — Repeatability and reproducibility contingency tables. (PDF) [file pone.0032800.s004.pdf]

Repeatability and reproducibility contingency tables

The following tables present a detailed breakdown of repeatability and reproducibility results. Table S4a presents test-retest results for each of 1,666 pairs of decisions. Table S4b presents all pairwise combinations of decisions on 718 image pairs by two examiners.

(NONMATES)		Retest								
		NV	VEO			VID			Total	
Initial Test			Excl	Inconc	Indiv	Excl	Inconc	Indiv		
NV		36	2	7	0	3	3	0	51	7.9%
VEO	Exclusion	2	13	6	0	15	1	0	37	5.7%
	Inconclusive	14	5	27	0	13	8	0	67	10.3%
	Individualization	0	0	0	0	0	0	0	0	0.0%
VID	Exclusion	3	7	8	0	391	24	0	433	66.8%
	Inconclusive	1	0	7	0	24	25	0	57	8.8%
	Individualization	0	0	0	0	2	1	0	3	0.5%
	Total	56	27	55	0	448	62	0	648	100.0%

(MATES)		Retest								
		NV	VEO			VID			Total	
Initial Test			Excl	Inconc	Indiv	Excl	Inconc	Indiv		
NV		215	2	24	0	1	2	1	245	24.1%
VEO	Exclusion	8	20	20	0	3	1	3	55	5.4%
	Inconclusive	22	6	78	1	3	26	11	147	14.4%
	Individualization	0	0	1	0	0	0	0	1	0.1%
VID	Exclusion	6	1	17	0	44	59	44	171	16.8%
	Inconclusive	4	1	19	2	2	85	21	134	13.2%
	Individualization	0	0	7	1	9	12	236	265	26.0%
	Total	255	30	166	4	62	185	316	1,018	100.0%

Table S4a: Intra-examiner repeatability. Test-retest results for the 72 retest participants. Data for mated pairs combines results from *RandomMates* and *FalseNeg* datasets with the effect of over-sampling false negatives: VEO exclusions constituted 1.5% of initial test decisions; VID exclusions, 4.8%. Data for nonmated pairs combines the results from *RandomNonMates* and *FalsePos* datasets.

(NONMATES)		Examiner 2								
		NV	VEO			VID			Total	
Examiner 1			Excl	Inconc	Indiv	Excl	Inconc	Indiv		
NV		910	149	432	0	192	120	0	1,803	9.5%
VEO	Exclusion	149	260	166	0	552	49	0	1,176	6.2%
	Inconclusive	432	166	678	0	481	180	0	1,937	10.2%
	Individualization	0	0	0	0	0	0	0	0	0.0%
VID	Exclusion	192	552	481	0	10,552	757	25	12,559	66.0%
	Inconclusive	120	49	180	0	757	414	3	1,523	8.0%
	Individualization	0	0	0	0	25	3	0	28	0.1%
	Total	1,803	1,176	1,937	0	12,559	1,523	28	19,026	100.0%

(MATES)		Examiner 2								
		NV	VEO			VID			Total	
Examiner 1			Excl	Inconc	Indiv	Excl	Inconc	Indiv		
NV		11,064	242	2,348	8	139	483	170	14,454	28.1%
VEO	Exclusion	242	116	218	1	44	77	50	748	1.5%
	Inconclusive	2,348	218	3,696	44	408	1,371	1,005	9,090	17.7%
	Individualization	8	1	44	2	2	25	58	140	0.3%
VID	Exclusion	139	44	408	2	340	748	765	2,446	4.8%
	Inconclusive	483	77	1,371	25	748	4,674	1,302	8,680	16.9%
	Individualization	170	50	1,005	58	765	1,302	12,472	15,822	30.8%
	Total	14,454	748	9,090	140	2,446	8,680	15,822	51,380	100.0%

Table S4b: Inter-examiner reproducibility. Counts of all pairwise combinations of decisions; limited to pairs of retest participants responding to the same image pairs as on the initial test.

(MATES)		Multi42								
		NV	VEO			VID			Total	
Initial Test			Excl	Inconc	Indiv	Excl	Inconc	Indiv		
NV		–	–	–	–	–	–	–	–	–
VEO	Exclusion	10	4	8	0	1	1	0	24	22.9%
	Inconclusive	–	–	–	–	–	–	–	–	–
	Individualization	–	–	–	–	–	–	–	–	–
VID	Exclusion	6	1	9	0	23	19	23	81	77.1%
	Inconclusive	–	–	–	–	–	–	–	–	–
	Individualization	–	–	–	–	–	–	–	–	–
	Total	16	5	17	0	24	20	23	105	100.0%

Table S4c: Intra-examiner repeatability results from the *Multi42* dataset for mated data. Test-retest results for 42 examiners (group B) on *FalseNeg_M* dataset. *Nonmate* repeatability results for *Multi42* are limited to a single data point (initial test: VID individualization; retest: VEO inconclusive).
