# Supplementary material for: Repeatability and Reproducibility of Decisions by Latent Fingerprint Examiners
Source: PLoS One. 2012 Mar 12;7(3):e32800. doi: 10.1371/journal.pone.0032800 (PMC3299696; doi:10.1371/journal.pone.0032800)
Supplement: Information S5 — Within-test repeatability of value decisions. (PDF) [file pone.0032800.s005.pdf]

Within-test repeatability of value decisions

The across-test repeatability results are based on repeat presentations of test questions separated by an interval of approximately seven months. The elapsed time for *Within-test* repeatability results (latent value decisions only) varied widely: an examiner might have encountered the repeat latent within one hour of the first presentation, or weeks later. The elapsed time depended on the random sequencing of test questions and the pace at which each examiner proceeded through the test.

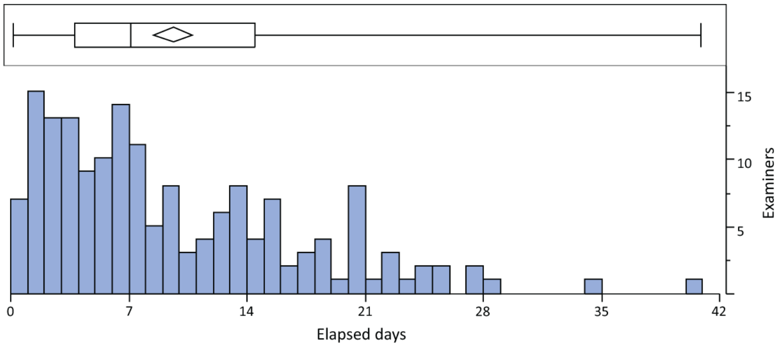

Fig. S5a: Number of elapsed days from start to finish of the initial test by examiner (median 7.2). These numbers are based on timestamps recorded in a log file and may not be highly reliable. Six extreme and implausible outliers were excluded (likely clock issues involving a shared workstation).

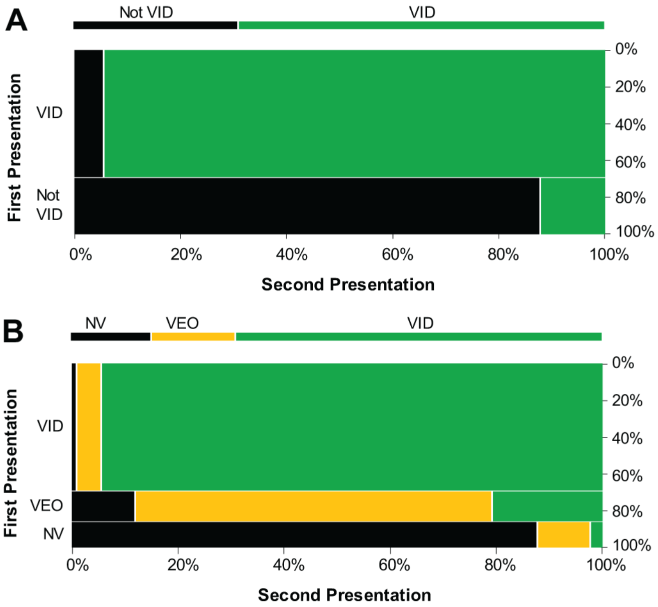

Fig. S5b: Short-term (*Within-test* dataset) repeatability of latent value decisions. (A) 2-way latent value decisions; $\overline{P}$ = 92.2%. (B) 3-way latent value decisions including category "value for exclusion only"; $\overline{P}$ = 88.8%. (Compare with Fig. 1 and Table 1, which show test-retest repeatability.)

	Second Presentation				
First Presentation	NV	VEO	VID	Total	Repeated
NV	113	13	3	129	88%
VEO	18	100	31	149	67%
VID	7	29	586	622	94%
Total	138	142	620	900	

Table S5c: Short-term (*Within-test* dataset) repeatability of latent value decisions. Value decision data for Fig. S5b. (Compare with Fig. 1 and Table 1, which show test-retest repeatability.)
