# Supplementary material for: Repeatability and Reproducibility of Decisions by Latent Fingerprint Examiners
Source: PLoS One. 2012 Mar 12;7(3):e32800. doi: 10.1371/journal.pone.0032800 (PMC3299696; doi:10.1371/journal.pone.0032800)
Supplement: Information S6 — Data used in the latent value repeatability analysis. (PDF) [file pone.0032800.s006.pdf]

Data used in the latent value repeatability analysis

When a latent was presented to an examiner more than once during the initial test or during the repeatability test, the examiner's first latent value decision on each test was used in this analysis.

The datasets (*RandomMates* and *RandomNonMates*) on which the across-test latent value statistics are based do not include any reassignments of mated image pairs on which the examiners initially committed false negative errors. We therefore consider whether this exclusion might bias our latent value repeatability results. Although the conditional probabilities for each row of Table S6a differ somewhat from those in Table 1 (in the main paper), we conclude that these differences would not have a substantial biasing effect given that these data (*FalseNeg* dataset) represent only 5.3% of the original test mix.

	Retest				
Initial Test	NV	VEO	VID	Total	Repeated
VEO	8	40	7	55	72%
VID	6	18	147	171	86%
Total	14	58	154	226	

Table S6a: Repeatability of latent value decisions on *FalseNeg* dataset; $\overline{P}$ = 82.7% (as compared to 3-way $\overline{P}$ = 84.6% for the *RandomMates* dataset).
