# Supplementary material for: Repeatability and Reproducibility of Decisions by Latent Fingerprint Examiners
Source: PLoS One. 2012 Mar 12;7(3):e32800. doi: 10.1371/journal.pone.0032800 (PMC3299696; doi:10.1371/journal.pone.0032800)
Supplement: Information S7 — Difficulty predicts repeatability and reproducibility. (PDF) [file pone.0032800.s007.pdf]

Difficulty predicts repeatability and reproducibility

Examiners were asked to indicate the difficulty of each comparison performed on a scale from "obvious" to "very difficult". Fig. S7a shows the association between examiner ratings of difficulty and the repeatability (chart A) and reproducibility (chart B) of exclusion decisions. These charts, which are analogous to those in Fig. 4 for individualization decisions, represent estimated posterior probabilities of decisions being repeated or reproduced given the examiner's assessment of difficulty and what the decision was.

Chart B (reproducibility) is constructed from all unordered pairs of examiner responses where the initial decision is exclusion, and the two examiners are distinct. Each exclusion decision therefore contributes equally to the distribution of difficulties for examiner 1. The mix of examiner 2 decisions is influenced more by those image pairs that were excluded by many examiners than by image pairs that were excluded by few examiners.

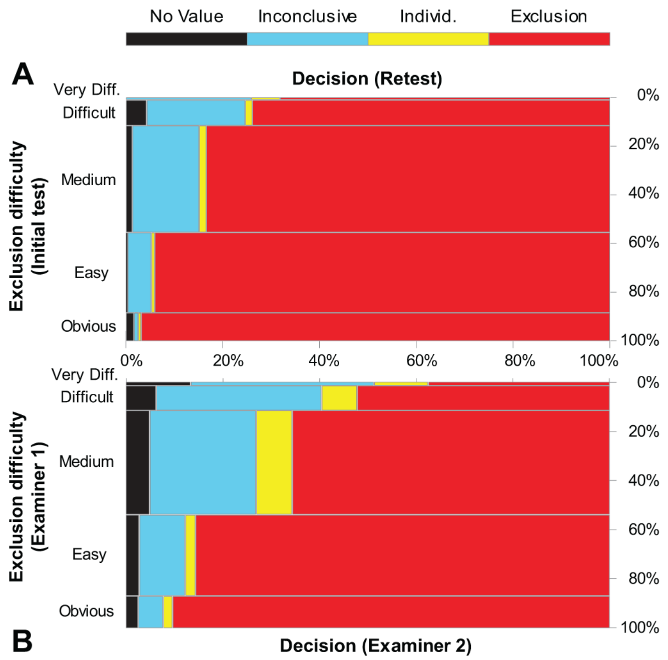

Fig. S7a: Repeatability (A) and reproducibility (B) of exclusion decisions by difficulty. (A) Retest decisions by difficulty where the initial test decision was an exclusion: 696 initial exclusion decisions (each retested) on 309 image pairs, 176 of which were nonmated, 133 mated. (B) Reproducibility of exclusion decisions by difficulty: 1,830 exclusion decisions (16,929 paired examiner responses) by the 72 examiners on 376 image pairs, 208 of which were nonmated pairs, 168 mated. In Chart A the 696 decisions (470 *RandomNonMates* and 226 *FalseNegs*) were weighted to correct for the disproportionate number of false negative errors that were deliberately included in the retest (for an effective sample size of 498 decisions).
