# Supplementary material for: Repeatability and Reproducibility of Decisions by Latent Fingerprint Examiners
Source: PLoS One. 2012 Mar 12;7(3):e32800. doi: 10.1371/journal.pone.0032800 (PMC3299696; doi:10.1371/journal.pone.0032800)
Supplement: Information S8 — Summary agreement statistics. (PDF) [file pone.0032800.s008.pdf]

Summary agreement statistics

This section provides a tabular summary of our key results, including statistics using both percentage agreement ($\overline{P}$) and *kappa*$_N$, a multi-rater, chance-adjusted index of agreement. *Kappa*$_N$ is closely related to the commonly-used Fleiss' *kappa* [1], which is defined as follows:

$$kappa = \frac{(\overline{P} - \overline{P}_e)}{(1 - \overline{P}_e)}$$

where

$$\overline{P}_e = \sum_{j=1}^{k} P_j^2 \ ,$$

$$P_j = \frac{1}{Nn} \sum_{i=1}^{N} n_{ij} \ ,$$

and N is the number of test questions (images or image pairs), n is the number of decisions, k is the number of decision categories, and n_{ij} is the number of decisions assigning the ith image (or image pair) to the jth category.

Implicit in this definition of chance, however, is the assumption that the marginal distributions are fixed. In our study, examiners had no prior knowledge of the prevalence of data by categories (e.g., proportions of value and no value images, mated and unmated pairs). Therefore we do not reduce the observed agreement according to the marginals (P_j), because those marginals themselves represent agreement among the examiners about prevalence. Instead, we opt to use the free marginal solution suggested by Brennan and Prediger ([2]; see also [3, 4, 5]):

$$kappa_N = \frac{(\overline{P} - 1/k)}{(1 - 1/k)}$$

Kappa$_N$ ranges from -1 to 1: a value of 1 indicates that examiners are in complete agreement; 0 indicates the level of agreement expected by chance; and a negative value indicates that the raters agreed less often than would be expected by chance.

Both $\overline{P}$ and *kappa*$_N$ implicitly treat all disagreements as being equally serious. So, for example, the disagreement "individualization vs. exclusion" is not weighted differently than the disagreement "individualization vs. inconclusive;" we therefore report separate statistics for various types of disagreements.

In the following tables, inter-examiner (reproducibility) statistics are computed from the initial test results, limited to the 72 retest participants.

Label	Description	Classes	Repeatability Intra $\overline{P}$	Repeatability Intra $Kappa_N$	Reproducibility Inter $\overline{P}$	Reproducibility Inter $Kappa_N$
2-way value, Retest (Fig. 1,Fig. 5)	Datasets: *RandomMates* & *RandomNonMates* n_{INTRA} = 1,403 decisions (339 latents)	VID, Not VID	0.897	0.795	n/a	n/a
3-way value, Retest (Fig. 1,Fig. 5)	Datasets: *RandomMates* & *RandomNonMates* n_{INTRA} = 1,403 decisions (339 latents)	VID, VEO, NV	0.846	0.769	n/a	n/a
2-way value, Within test (Fig. S4,Fig. 5)	Dataset: *Within-test* n_{INTRA} = 306 decisions (104 latents) n_{INTER} = 6,890 decisions (350 latents)	VID, Not VID	0.918	0.837	0.843	0.686
3-way value, Within test (Fig. S4,Fig. 5)	Dataset: *Within-test* n_{INTRA} = 306 decisions (104 latents) n_{INTER} = 6,890 decisions (350 latents)	VID, VEO, NV	0.876	0.814	0.757	0.635
2-way value, false negatives only	Dataset: *FalseNeg* n_{INTRA} = 226 decisions (112 latents)	VID, Not VID	0.863	0.726	n/a	n/a
3-way value, false negatives only	Dataset: *FalseNeg* n_{INTRA} = 226 decisions (112 latents)	VID, VEO, NV	0.827	0.741	n/a	n/a
2-way value, Retest	Datasets: *RandomMates* & *RandomNonMates* n_{INTRA} = 842 decisions (197 latents on which examiners were not unanimous on VID)	VID, Not VID	0.833	0.665	n/a	n/a
2-way value Within test	Dataset: *Within-test* n_{INTRA} = 190 decisions n_{INTER} = 842 decisions (197 latents on which examiners were not unanimous on VID)	VID, Not VID	0.868	0.737	0.752	0.505

Table S8a: Agreement statistics for latent value decisions. These statistics are calculated over various subsets of the test data (limited to the 72 retest participants). The retest yielded 1,403 pairs of intra-examiner latent value decisions among randomly selected latents (*RandomMates* and *RandomNonMates* datasets) that were assigned to an examiner on the initial test and on retest. When a latent was presented to an examiner more than once during the initial test or during the repeatability test, the examiner's first latent value decision on each test was used in this analysis. Among the 900 second presentations of a latent to the same examiner during the initial test, 306 of these were assigned to the 72 retest participants (104 distinct latents). Retest participants made 6,890 latent value decisions (on the initial test (counting only the first presentation of a latent to each participant). The *FalseNeg* dataset yielded 226 pairs of latent value decisions (initial test and retest). On the initial test, examiners were not unanimous in their latent value decisions (VID vs. Not VID) on 197 of the 356 latents. The retest included 842 second decisions on these 197 latents.

Label	Description	Classes	Repeatability Intra $\overline{P}$	Repeatability Intra *Kappa*$_N$	Reproducibility Inter $\overline{P}$	Reproducibility Inter *Kappa*$_N$
2-way comparison<br>Mates (false negatives)	Exclusions	Any exclusion (error)<br>Not excluded	0.970	0.939	0.899	0.798
2-way comparison<br>Mates (true positives)	VID individualizations	VID individualization<br>Other	0.922	0.843	0.866	0.732
3-way comparison<br>Mates	Three basic comparison classes	VID individualization<br>VEO exclusion (error)<br>Other	0.903	0.854	0.798	0.696
7-way comparison<br>Mates	Full comparison detail	VID individualization<br>VID exclusion (error)<br>VID inconclusive<br>VEO individualization<br>VEO exclusion (error)<br>VEO inconclusive<br>NV	0.775	0.738	0.632	0.570

Table S8b: Mate comparisons (supporting data for Fig. 6 and Fig. 7). Repeatability statistics based on *RandomMates* dataset (n_{INTRA} = 389 mates, 792 decisions; by 72 examiners). The *RandomMates* dataset includes mates that resulted in false negative errors on the initial test, but no mated pair was reassigned to an examiner who made a false negative error on that pair on the initial test. These repeatability statistics are therefore biased estimators of repeatability on mated pairs: they slightly overestimate mate repeatability. Reproducibility statistics are based on initial test results (n_{INTER} = 520 mates, 5,134 decisions; limited to the 72 examiners who participated in the retest).

Label	Description	Classes	Repeatability Intra $\overline{P}$	Repeatability Intra *Kappa*$_N$	Reproducibility Inter $\overline{P}$	Reproducibility Inter *Kappa*$_N$
2-way comparison<br>Nonmates<br>(false positives)	Individualizations	Any individualization (error)<br>Not individualized	1	1	0.997	0.995
2-way comparison<br>Nonmates<br>(true negatives)	Exclusions	Any exclusion<br>Not excluded	0.859	0.718	0.796	0.592
3-way comparison<br>Nonmates	Three basic comparison classes	VID individualization (error)<br>VEO exclusion<br>Other	0.859	0.788	0.796	0.694
7-way comparison<br>Nonmates	Full comparison detail	VID individualization (error)<br>VID exclusion<br>VID inconclusive<br>VEO individualization (error)<br>VEO exclusion<br>VEO inconclusive<br>NV	0.763	0.723	0.666	0.611

Table S8c: Nonmate comparisons (supporting data for Fig. 6 and Fig. 7). Repeatability statistics based on *RandomNonMates* dataset (n_{INTRA} = 210 nonmates, 645 decisions; by 72 examiners). The *RandomNonMates* dataset was constrained so that none of the three examiners who made false positive errors on the initial test were reassigned those image pairs on the retest; the biasing effect is negligible. Reproducibility statistics are based on initial test results (n_{INTER} = 219 nonmates, 2,066 decisions; limited to the 72 examiners who participated in the retest).

Label	Description	Classes	Repeatability Intra $\overline{P}$	Repeatability Intra $Kappa_N$	Reproducibility Inter $\overline{P}$	Reproducibility Inter $Kappa_N$
2-way false negatives (see Table 3)	Exclusions	Any exclusion (error)<br>Not excluded	0.301	-0.398	0.647	0.293

Table S8d: False negative errors. Repeatability statistics based on the *FalseNeg* dataset (n_{INTRA} = 133 mates, 226 decisions by 64 examiners). $Kappa_N$ is negative because the majority of these decisions were changed (not excluded on the retest). The reproducibility statistics were calculated by weighting each image pair according to the number of times it was reassigned on the retest, to correspond to the same mix of comparisons, which includes many pairs that were assigned to more than one examiner (n_{INTER} = 133 mates, 2,472 decisions by 72 examiners).

Label	Description	Classes	Repeatability Intra $\overline{P}$	Repeatability Intra $Kappa_N$	Reproducibility Inter $\overline{P}$	Reproducibility Inter $Kappa_N$
2-way false positives (see Table 1)	Individualizations	VID individualization (error)<br>Other	1	n/a	0.902	0.804

Table S8e: False positive errors. Repeatability statistics based on *FalsePos* and *FalsePos_M* datasets (n_{INTRA} = 4 nonmates, 8 decisions by 4 examiners). Note that these repeatability statistics include one response from the *Multi42* dataset. Reproducibility statistics based on initial test results (n_{INTER} = 6 nonmates, 59 decisions by 72 examiners). The reproducibility statistics include data on all 6 nonmated pairs that resulted in false positive decisions, although only 4 of these decisions occurred among the retest participants.

References

1. Fleiss JL (1971) Measuring nominal scale agreement among many raters. Psychol Bull 76(5): 378-382. Available: http://www.wpic.pitt.edu/research/biometrics/Publications/Biometrics%20Archives%20PDF/395-1971%20Fleiss0001.pdf
2. Brennan RL, Prediger DJ (1981) Coefficient kappa: some uses, misuses, and alternatives. Educational and Psychological Measurement 41: 687-699.
3. Gwet K (2002) Kappa statistic is not satisfactory for assessing the extent of agreement between raters. Series: Statistical Methods for Inter-Rater Reliability Assessment 1(1):1-5. Available: http://www.agreestat.com/research_papers/kappa_statistic_is_not_satisfactory.pdf
4. Perrault WD, Jr, Leigh LE (1989) Reliability of nominal data based on qualitative judgments. J Marketing Res 26: 135-148. Available: http://www.viktoria.se/~dixi/BISON/resources/perreault-leigh-1989.pdf
5. Rust RT, Cooil B (1994) Reliability measures for qualitative data: theory and implications. J Marketing Res 31: 1-14. Available: http://www2.owen.vanderbilt.edu/bruce.cooil/Documents/Publications/JMR%201994.pdf
