# Supplementary material for: Repeatability and Reproducibility of Decisions by Latent Fingerprint Examiners
Source: PLoS One. 2012 Mar 12;7(3):e32800. doi: 10.1371/journal.pone.0032800 (PMC3299696; doi:10.1371/journal.pone.0032800)
Supplement: Information S9 — Confidence intervals. (PDF) [file pone.0032800.s009.pdf]

Confidence intervals

This section presents confidence intervals for several of the statistics presented in Fig. 5, Fig. 6, and Table 7. This information is included to address the question of whether the sample sizes are large enough to justify our interpretations of the data.

Here we use the beta-binomial distribution to model repeatability and reproducibility of examiner decisions [1]. This approach is applicable only to the binomial (2-way) decisions. It yields estimates that differ slightly from percentage agreement shown in Figs. 5 and 6.

Reproducibility is the probability that two examiners, looking at the same image-pair, will give the same response. Under the beta-binomial distribution, the probability that an image i classified (say) as VID, as opposed to Not VID, is given by an unobservable $p[i]$, where i is the image ID. $p[i]$ is assumed to be sampled from a beta distribution with mean p and variance $\delta p(1\text{-}p)$. The parameter δ is the "overdispersion" – excess variance that results from the real differences among the images. The probability that two examiners agree on a randomly selected image is then

$$P(agreement) = p^2 + (1-p)^2 + 2\delta p(1-p)$$

Overdispersion increases the probability of agreement.

The tool used to produce these estimates (JMP™) reports 95% likelihood profile confidence intervals for the two individual parameters of the beta-binomial distribution, p and δ. Conservative estimates of the 90% confidence intervals for reproducibility were then obtained by applying Bonferroni's inequality.

For repeatability, p is the probability of agreement (not the probability of a particular classification, as was the case for reproducibility). Hence, the confidence intervals reported for repeatability are the 95% likelihood profile intervals for p (Table S9a).

	Repeatability			Reproducibility		
	Estimate	Lower 95%	Upper 95%	Estimate	Lower 90%	Upper 90%
Latent value {VID vs. Not VID} (Days)	0.917	0.879	0.946			
Latent value {VID vs. Not VID}	0.895	0.873	0.914	0.846	0.824	0.869
Mates {VID Individualization vs. Other}	0.924	0.899	0.943	0.870	0.846	0.893
Nonmates {Exclusion vs. Other}	0.863	0.828	0.893	0.794	0.749	0.838

Table S9a: Estimates of repeatability and reproducibility of examiner decisions. Within-test repeatability ("Days") estimates are also provided for latent value decisions.

The reproducibility estimates in Table S9b (which correspond to those of Table 7) represent the conditional probability that a result (exclusion or non-exclusion) from a first examiner is confirmed by a second examiner, among mated image-pairs. The conditional probability estimates were derived from the estimated parameters of the beta-binomial distribution:

$$p + \delta(1-p)$$

In order to have a true conditional probability to estimate, we are assuming that the sample mated image-pairs have been randomly selected from a target population of such pairs, and that the examiners have been randomly selected from a target population. We are not claiming that the images or examiners are representative of casework; rather, the confidence intervals assume those populations of image pairs and examiners defined by the selection criteria used in this test. The confidence intervals also assume that the beta-binomial is the correct distribution for reproducibility.

	Repeatability (Retests)			Reproducibility (Initial Test)		
	Estimate	Lower 95%	Upper 95%	Estimate	Lower 90%	Upper 90%
FN (n=226)	0.301	0.253	0.353	0.192	0.151	0.243
Not FN (n=792)	0.970	0.958	0.978	0.945	0.932	0.956

Table S9b: Comparison of repeatability and reproducibility results, conditioned upon whether the initial decision was a false negative (FN) error; duplicates Table 7 with 90% confidence intervals. For comparability, all estimates are limited to responses of the retest participants. “Not FN” includes NV, inconclusive, and individualization.

References

1. Schuckers ME (2003) Using the beta-binomial distribution to assess performance of a biometric identification device. Int. J. Image Graph 3(3): 523-529.
