# Supplementary material for: Repeatability and Reproducibility of Decisions by Latent Fingerprint Examiners
Source: PLoS One. 2012 Mar 12;7(3):e32800. doi: 10.1371/journal.pone.0032800 (PMC3299696; doi:10.1371/journal.pone.0032800)
Supplement: Information S10 — Use of “Value for Exclusion Only” category. (PDF) [file pone.0032800.s010.pdf]

Use of “Value for Exclusion Only” category

Examiners participating in the initial study [1] were asked how prints “of value for exclusion only” are defined by the operating procedures that their agencies currently use: 55% make a 2-way distinction {VID, not VID} and do not differentiate between VEO and NV; 14% make a 2-way distinction {NV, not NV} and do not differentiate between VID and VEO; the remainder make a 3-way distinction {VID, VEO, NV}.

References

1. Ulery BT, Hicklin RA, Buscaglia J, Roberts MA (2011) Accuracy and reliability of forensic latent fingerprint decisions. Proc Natl Acad Sci USA 108(19): 7733-7738. Available: http://www.pnas.org/content/108/19/7733.full.pdf
